# The Role of Ceramides in Diabetes and Cardiovascular Disease Regulation of Ceramides by Adipokines

**DOI:** 10.3389/fendo.2020.569250

**Published:** 2020-10-02

**Authors:** Bianca C. Field, Ruth Gordillo, Philipp E. Scherer

**Affiliations:** ^1^Touchstone Diabetes Center, Department of Internal Medicine, University of Texas Southwestern Medical Center, Dallas, TX, United States; ^2^Department of Cell Biology, University of Texas Southwestern Medical Center, Dallas, TX, United States

**Keywords:** leptin, adiponectin, adiponectin receptors, sphingolipids, ceramidase, adipokine

## Abstract

Metabolic dysfunction is intertwined with the pathophysiology of both diabetes and cardiovascular disease. Recently, one particular lipid class has been shown to influence the development and sustainment of these diseases: ceramides. As a subtype of sphingolipids, these species are particularly central to many sphingolipid pathways. Increased levels of ceramides are known to correlate with impaired cardiovascular and metabolic health. Furthermore, the interaction between ceramides and adipokines, most notably adiponectin and leptin, appears to play a role in the pathophysiology of these conditions. Adiponectin appears to counteract the detrimental effects of elevated ceramides, largely through activation of the ceramidase activity of its receptors. Elevated ceramides appear to worsen leptin resistance, which is an important phenomenon in the pathophysiology of obesity and metabolic syndrome.

## Introduction

**Figure 1:**
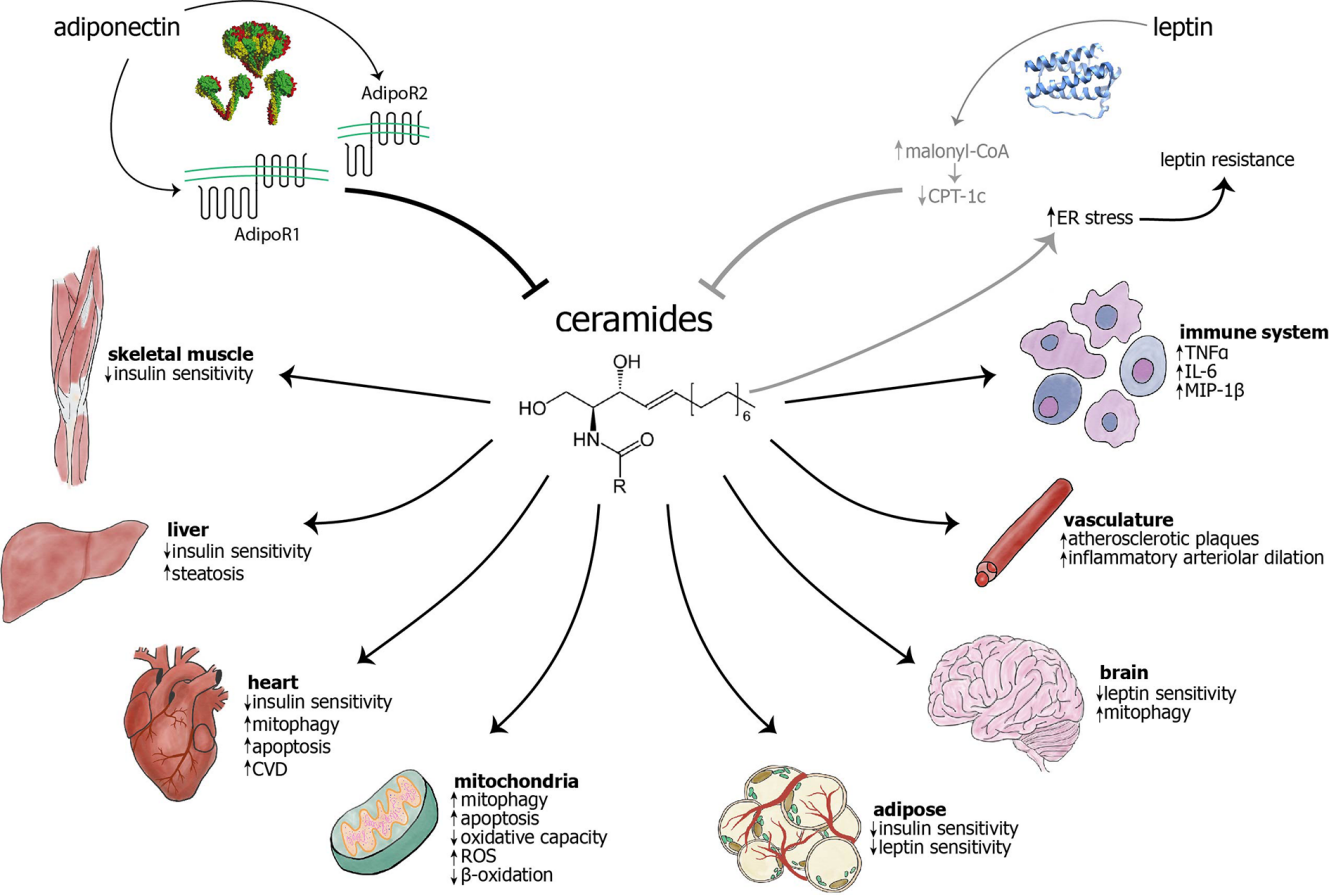
The adipokines adiponectin and leptin have largely inhibitory effects on ceramide levels, while ceramides are known to cause myriad effects in cellular components, different cell types, and organs. Adiponectin inhibits ceramides through the intrinsic ceramidase activity of its receptors (1), AdipoR1 and AdipoR2. The mechanism of leptin’s action on ceramides remains unknown, although it has been hypothesized that leptin may exert its influence through inhibition of carnitine palmitoyltransferase-1c (CPT-1c) by malonyl-CoA (2). Conversely, ceramide accumulation may cause ER stress and subsequent leptin resistance (3–6). The previous two relationships are visualized in gray to indicate that they have not been extensively studied and characterized. Ceramides are known to act on mitochondria and cause increased mitophagy, apoptosis, and generation of reactive oxygen species (ROS), as well as decreased β-oxidation and overall oxidative capacity. Ceramides have been shown to cause mitophagy in cardiomyocytes and neurons, among others (not shown) (7). Ceramides decrease insulin sensitivity in skeletal muscle, liver, heart, and adipose (8, 9). Their accumulation may cause decreased leptin sensitivity in the brain and adipose tissue. In the vasculature, ceramide accumulation leads to increased atherosclerotic plaques and inflammatory arteriolar dilation (10–13). They can also lead to increased cardiovascular disease (14, 15), hepatic steatosis (16), and increased inflammation in immune cells (17). As opposed to other targets, skin largely benefits from increased ceramides, with increased cell differentiation and wound healing, as well as decreased senescence (18, 19). AdipoR, adiponectin receptor; CPT-1c, carnitine palmitoyltranferase 1c; CVD, cardiovascular disease; ER, endoplasmic reticulum; IL-6, interleukin-6; MIP-1, macrophage inflammatory protein 1β; ROS, reactive oxygen species; TNFα, tumor necrosis factor α.

Sphingolipids are by far the most structurally diverse class of lipid molecules. The structural complexity of sphingolipids stems from variation in three components of these lipids: a long chain base, an amide-linked fatty acid, and a head group (20). They are signaling molecules involved in diverse cellular functions, from control of the cell cycle to degradation of plasma membrane proteins (21). They are involved in the most devastating inborn errors and degenerative diseases, including cancer, Alzheimer’s disease and dementia (22). In recent years, ceramides, a subgroup of sphingolipids, have received increased attention for their role in myriad pathophysiologic mechanisms, including those underlying cancer (23–25), inflammation (26), depression (27), and neurodegenerative disorders (28). Additionally, more recent studies have highlighted their increasingly important role in obesity and diabetes (9, 29–31) as well as cardiovascular disease (CVD) (32). Preventing aberrant ceramide deposition can prevent or ameliorate a variety of cardiometabolic pathologies, including insulin resistance, CVD (atherosclerosis, heart failure), and hepatic steatosis (33, 34), as well as mitochondrial dysfunction (**Figure 1**). Here, we will focus on ceramides’ role in diabetes and CVD, two of the leading causes of death in the United States (35) and worldwide (36).

## Introduction to Ceramides

### Ceramide Biochemistry

Ceramides constitute a simple subtype of sphingolipids composed of a variable-length fatty acid and an amino group (a sphingoid base, usually sphingosine). Their unique structure, highly hydrophobic with a small side chain, enable them to function well both as a structural membrane component and signaling molecule (20, 37). Under physiologic conditions, they range from short chain (C6:0) to very long chain (VLC) (C26:0), although longer chain ceramides are more commonly found in mammalian cells. Ceramide biosynthesis can occur through three distinct pathways: *de novo*, sphingomyelin hydrolysis, or sphingolipid salvage. *De novo* synthesis occurs in the endoplasmic reticulum (ER) and involves ceramide synthases (CerS) as well as serine palmitoyl-transferase (SPT), which catalyzes the rate-limiting step and is inhibited by the drug myriocin. In another pathway, sphingomyelinase (SMase) hydrolyzes sphingomyelin to form ceramide. Finally, in the salvage pathway, alkaline ceramidase (ACER), which is inhibited by the drug D-erythro-MAPP, and acid ceramidase (AC) work in the endolysosomal system to regenerate ceramide from sphingosine (38, 39).

Sphingomyelinases and ceramidases exist in three forms: acid, neutral, and alkaline. Their subtype depends on their subcellular localization, function, and optimal pH. Generally, acid SMases and ceramidases function in lysosomes, neutral enzymes along the plasma membrane, and alkaline enzymes in the Golgi or ER (40). Furthermore, CerSs, important in both *de novo* and salvage pathways, preferentially catalyze certain fatty acyl-CoA substrates in order to make different ceramide subtypes (39, 40). Because ceramides are the center of a web of sphingolipid biosynthesis reactions, it can be difficult to prove causation between particular metabolites and enzymes (38).

Dihydroceramides are the precursor metabolites of ceramides in the *de novo* sphingolipid biosynthesis pathway. CerS form dihydroceramides by condensing sphinganine and acyl-CoA, and they are a family of six isoforms (CerS1-6) that each display specificity for certain acyl-CoA chain lengths. Ceramides are formed from dihydroceramides by the action of the enzyme dihydroceramide desaturase (41, 42).

### Ceramides as Cell Membrane Components

Ceramides are key to the structural integrity the plasma and mitochondrial membranes. While they are restricted to the membrane in which they are formed, they can flip-flop between membrane leaflets (43), although this movement occurs slowly (44). This incorporation can change membrane configuration and relative hydrophobicity of the plasma membrane-cytosol interface, changing the affinity for the membrane of a number of proteins, which in turn affect the activity of membrane enzymes (45).

### Ceramides in Circulation

Clinical studies show that circulating ceramides primarily originate in the liver, a major site of *de novo* ceramide synthesis (17, 46), although adipocytes may also contribute to circulating ceramides in some species (33). They circulate at a concentration of about 10μM and are primarily transported in circulation by lipoproteins (17, 46, 47), while exosomal transport may also significantly contribute (48). One study, using thin layer and high-performance liquid chromatography (TLC and HPLC, respectively), found that plasma ceramides are primarily (~80%) carried by very-low-density lipoprotein (VLDL) and low-density lipoprotein (LDL), with the remaining ceramide being carried by albumin (~15%) and high-density lipoprotein (HDL; ~5%) (46). Another study, using fast performance liquid chromatography (FPLC), separated lipoprotein fractions and found that ceramides were primarily carried by LDL (60.3 +/−6.7%) and to a lesser extent by HDL (24.1+/−7.4%), and VLDL (15.6+/−9.9%). However, ceramides comprised a small fraction of total lipids carried by these lipoproteins (LDL and VLDL ~0.6%; HDL ~0.1%). While the distribution of ceramide species carried by these lipoprotein classes were largely similar, LDL and VLDL carried more similar proportions of specific ceramide species (less C16:0, more C24:0), while HDL carried others (more C16:0, less C24:0) (47). Additionally, recent research has found that ceramide species are also transported in exosomes from adipocytes and endothelial cells (48).

### Ceramides in Mitochondria

Ceramides are a normal component of mitochondrial membranes, and they are necessary for normal mitochondrial function. Purified mitochondria and mitochondria-associated membranes have been shown to generate ceramides *in vitro*, and specific ceramide synthases (CerS2, CerS4, and CerS6) are present in the inner and outer mitochondrial membranes (49). Furthermore, normal biological mechanisms can be mediated by ceramides. For instance, CerS6-generated ceramides may mediate pruning of oligodendrocytes during normal brain development (50).

While ceramides are necessary for normal mitochondrial functions, accumulation of excess ceramides have been shown to induce mitochondrial dysfunction (51, 52). Increased mitochondrial ceramide content has been shown to have numerous deleterious effects, including inhibition of mitochondrial oxidative capacity, depletion of ATP, inhibition of the electron transport chain, and enhanced generation of reactive oxygen species (51, 53–55). Additionally, studies show that ceramides can alter or nearly eliminate the respiration-dependent mitochondrial membrane potential (51, 56, 57). They disrupt the normal structure of the inner and outer mitochondrial membranes, increasing their permeability independent of cytochrome c and electron transfer activity (51). One study in a mouse model of ischemia showed that ceramide accumulation leads to inhibition of the respiratory chain (58). In mouse models of diet-induced obesity (DIO), pharmacologic or genetic depletion of ceramides increases mitochondrial complex activity [complex IV in subcutaneous white adipose tissue (sWAT), complexes II and IV in brown adipose tissue (BAT)], as well as respiration in sWAT, BAT, and brain (59). Pharmacological depletion of ceramides also decreased the respiratory exchange ratio (RER) in models of DIO (60). Several ceramide lipid storage diseases, including Fabry disease and neuronal ceroid lipofuscinosis, are associated with dysfunction in the mitochondrial respiratory chain (7). Other sphingolipid storage diseases cause inhibition of mitochondrial biogenesis (61).

### Ceramides in Mitophagy and Apoptosis

Beyond causing mitochondrial dysfunction, ceramides are known to regulate mitophagy (62). Accumulation of ceramide species in yeast led to increased mitochondrial fission and mitophagy (63). Similarly, in mouse and human cardiomyocytes, VLC ceramides produced by CerS2 led to increased mitophagy, along with increased oxidative stress and mitochondrial dysfunction (56). Ceramide-induced mitophagy can also help explain some pathophysiology and treatment mechanisms. For example, one study found that cigarette smoke caused mitophagy through increased levels of C16 ceramide, triggering necroptosis (64). Another study found that in glioblastoma cells, a plant agglutinin that is used to inhibit glioblastoma growth induced mitophagy-dependent cell death *via* increased ceramides (65).

Ceramides also have an established role in the induction of apoptosis (66), and their effects on mitochondria appear to be important in this process (52, 67). Apoptotic stimuli activate CerS in the mitochondrial membranes, indicating a role for ceramides in mitochondria-induced apoptosis (53). Mitochondria can function as sensors for cellular stress by monitoring local levels of specific lipids, including sphingolipids (45). This function facilitates the intrinsic apoptotic pathway. Formation of ceramide channels on the outer mitochondrial membrane leads to apoptosis, with ceramide species C16, C18, and C20 being most important in this process (52). Ceramides also work synergistically with the apoptotic protein Bax to induce mitochondrial outer membrane permeabilization in yeast (55). Furthermore, C2 ceramide was found to alter mitochondrial membrane potential and cause mitochondrial fragmentation, leading to apoptosis in cultured cardiomyocytes (57). Some interventions, including overexpression of Bcl-2 (68–71) and pharmacological inhibition of mitochondrial membrane depolarization (72, 73), have also prevented ceramide-induced apoptosis through prevention of mitochondrial dysfunction (74).

Ceramides may need to localize specifically to mitochondria to induce apoptosis. One group found that increased ceramides due to sphingomyelinase overexpression only induced apoptosis if the enzyme was targeted to mitochondria, but not if it was targeted to other cell compartments, including the inner plasma membrane, cytoplasm, ER, Golgi, and nucleus. Despite a resultant increase in whole-cell ceramide content, there was no induction of apoptosis when increased ceramides were targeted to these compartments (75). Furthermore, another group found that specific ceramide enzymes localize to the inner and outer membranes, respectively, suggesting that there exist distinct pools of ceramides (76).

### Ceramides in Metabolic Syndrome

Development of insulin resistance is a key step in the progression of metabolic syndrome, obesity, and diabetes, and it occurs when increasing levels of insulin are required to stimulate insulin-induced glucose uptake, particularly in myocytes and adipocytes. Studies have shown that plasma, adipose, and skeletal muscle ceramides are elevated in patients with obesity and type 2 diabetes mellitus (T2DM) (17, 77, 78), as well as in adipose, hepatic, skeletal muscle, and plasma samples from animal models of insulin resistance (17, 79–81). Moreover, ceramide levels in plasma, adipose, liver, and skeletal muscle negatively correlate with severity of insulin sensitivity in humans and in animal models (17, 77, 78, 81, 82). This is supported by the evidence that ceramides decrease the activity of Akt, which is a downstream effector of insulin signaling as well as of apoptosis and cell proliferation (24). Furthermore, adipose ceramide levels are higher in patients with increased hepatic lipid accumulation than in those with healthy livers (77). Reduced ceramide content prevents lipotoxicity induced by ceramide accumulation, preserving insulin and glucagon secretion (83). Ceramide bound to LDL also increases insulin resistance in skeletal myocytes and increases expression of inflammatory genes in macrophages, including IL-6 and TNFα (17). Clinical studies further prove that skeletal muscle ceramide content negatively correlates with insulin sensitivity (82, 84).

Relative amounts of particular ceramide species in insulin resistant states vary among sources. In one clinical study, skeletal muscle ceramides C16:0 and C18:3 were most strongly negatively correlated with insulin sensitivity (82). However, another clinical study showed that plasma C18:0 was most increased in obese T2DM participants over their healthy controls, but C24:0 was most abundant (78). More research is needed to clarify the relative impacts of specific ceramide species on insulin resistance.

Work by Summers and colleagues shows that ceramide production is an important factor in the development of insulin resistance through glucocorticoids and saturated fatty acids, but not through unsaturated fatty acids (16, 85). Glucocorticoids induce production of sphingolipid metabolites by promoting expression of the enzymes AC and glucosylceramide synthase (GCS), which produce ceramides through the salvage pathway. While Holland et al. did not measure or report an increase in acid sphingomyelinase activity, Cinque et al. demonstrated that dexamethasone, a glucocorticoid, induced thymocyte apoptosis by sequential activation of nuclear phosphoinositide specific phospholipase C-beta (PI-PLCβ) and acid sphingomyelinase (aSMase). Therefore, increased activity of both AC and aSMase, induced by glucocorticoids, could cause increased ceramide levels (86, 87). Saturated fatty acids promote ceramide accumulation in liver and skeletal muscle, as well as induction of insulin resistance (16). Conversely, reduction of ceramides has been shown to reverse the metabolic consequences of ceramide deposition in tissues. Induced overexpression of AC in mice, under the control of the adiponectin promoter, led to decreased ceramides in visceral and subcutaneous fat, liver, and serum, particularly ceramide species C16:0 and C18:0 (16, 88). After induction of AC expression, mice had reduced hepatic steatosis and improved insulin signaling and glucose metabolism, as shown by improvements in oral glucose tolerance tests and insulin tolerance tests, as well as increased glucose infusion rates and decreased hepatic glucose output during hyperinsulinemic-euglycemic clamps (88).

Ceramides’ induction of mitochondrial dysfunction appears to play a role in the development of metabolic syndrome and its associated conditions. Several studies specifically implicated ceramides produced by the enzyme ceramide synthase 6 (CerS6). One group found that ob/ob mice, which congenitally lack leptin expression, have elevated CerS6 in their liver, BAT, and sWAT, but not gonadal white adipose tissue (gWAT) (89), and another found that C16:0 ceramide from CerS6 inhibits mitochondrial beta-oxidation in BAT and liver (90). Another study found that in mice with high-fat diet (HFD)–induced weight gain, only CerS6 increased in mitochondria and mitochondria-associated membranes, not ceramide synthase 5 (CerS5). CerS6 also interacts with mitochondrial fission factor (MFF) to promote mitochondrial fission, and knockout of either of these proteins normalizes mitochondrial structure altered by HFD (91). In ob/ob and DIO mice, but not in db/db mice, which lack the leptin receptor, Raichur et al. found that these mice have elevated C16:0 ceramide in liver and plasma. They also found that these ablation of CerS6 and subsequent lowering of C16:0 ceramide by 50% in ob/ob and DIO mice corrected high blood glucose and insulin resistance, and also caused 25% weight loss without a change in food intake (89).

Evidence also points to roles for other CerS in metabolic dysfunction. In diabetic cardiomyopathy, ceramide synthase 2 (CerS2)- and CerS5-derived ceramides, including VLC ceramides, led to increased mitophagy and insulin resistance (56). In DIO mice, ceramide synthase 1 (CerS1) and C18:0 are elevated in skeletal muscle, and knockout of CerS1 globally as well as specifically from skeletal muscle improves insulin sensitivity through increased fibroblast growth factor 21 (FGF21) in muscle (92). The role of ceramides in metabolic syndrome will be discussed further in later sections examining the interaction of ceramides with adiponectin and leptin, respectively.

### Ceramides in Cardiovascular Disease

Ceramides have a well-established role in the development of CVDs, including atherosclerosis (15, 93), cardiomyopathy (15, 94, 95), heart failure (15, 96), myocardial infarction (14, 15), and stroke (14, 15). In particular, dihydroceramides, the precursors of ceramides in the *de novo* biosynthetic pathway of sphingolipids, are implicated in the development of CVD (15).

A substantial body of literature in rodent models of CVD indicates that ceramides are not only biomarkers of cardiovascular health, but also likely play a causative role in metabolic and CVD (8, 29, 94, 97–100). Studies in rodent models reveal that pharmacological inhibition of ceramide synthesis prevents heart failure secondary to ischemic cardiomyopathy and also prevents ventricular remodeling, fibrosis, and macrophage infiltration following myocardial infarction (94, 97–99). Moreover, such ceramide-lowering interventions also resolve many conditions underlying CVDs, including dyslipidemia, insulin resistance, hypertension, atherosclerosis, and hepatic steatosis (10–12, 16, 101–104). Manipulations of the *de novo* ceramide synthesis pathway further suggest that certain ceramide species are deleterious, whereas others are benign or beneficial (90, 92, 105, 106). Those containing the C16 or C18 acyl chain (90, 92, 105) and a double bond (i.e., ceramides, not dihydroceramides) (101) in the sphingolipid backbone are particularly harmful. Finally, studies in rodents reveal that ceramide degradation is a primary means by which adiponectin receptors, which have ceramidase activity (1), exert their antidiabetic, cardioprotective, and insulin-sensitizing actions (1, 107, 108). Cumulatively, these data identify ceramides as some of the more toxic metabolites that accumulate in metabolic distress.

Other authors have reported the existence of a strong correlation between CVD and increased dihydroceramide levels. Both dihydroceramides and ceramides correlate with the release of the inflammatory cytokine interleukin 6 (IL-6), but only dihydroceramides correlates with macrophage inflammatory protein 1β (MIP-1β) release (13, 15). Studies have also shown that dihydroceramide levels are elevated in patients with rheumatoid arthritis (109), patients with left ventricular assist devices (110) and hypertensive rats (13–15, 41, 111, 112).

Elevated levels of dihydroceramides have been found in atherosclerotic plaques (13) as well as in models of brain hypoxia (111, 112). What role this increase in dihydroceramides plays in plaque stability is still debatable, since the extracellular addition of dihydroceramides to human aortic smooth muscle cells did not cause apoptosis, whereas addition of ceramides did (13). After stroke, a key component of tissue injury results from ischemia and subsequent reperfusion, during which ceramides increase in mitochondria and the ER due to *de novo* synthesis (58, 74). Myocardial biopsies from patients with heart failure reveal increased levels of ceramide content. A common treatment for heart failure is implantation of a left ventricular assist device, which compensates for defects due to heart failure, leading to a reduction in mechanical load on the cardiac tissue. This intervention both lowered myocardial ceramide levels and improved whole-body and cardiac insulin sensitivity (113).

A meta-analysis of three prospective clinical studies in Finland, Switzerland, and Norway that followed patients with CVD found that ceramide levels, particularly certain ceramide ratios, were significantly positively correlated with cardiovascular death, independent of other lipid markers, including cholesterol, and C-reactive protein (114). It also found that ratios of long-chain ceramide species (C18:1/C16:0 and C18:1/C18:0) were more strongly correlated with negative cardiovascular outcomes than ratios of VLC ceramide species (C18:1/C24:0) were (114). Furthermore, ratios of ceramide species were shown to be more significant than gross changes (114, 115). Laaksonen et al. found that the ratios of (C18:1/C24:0)/(C18:1/C16:0) and (C18:1/C24:0)/(C18:1/C16:0) negatively correlate with coronary heart disease (CHD) and mortality. Another more recent study found that serum sphingolipids are markers of coronary artery disease, independent of cholesterol (100), further solidifying the strong correlation between these species and negative cardiac outcomes. Thus, plasma ceramide levels can be used as clinical biomarkers to predict risk of cardiovascular death.

Ceramides are also strongly implicated in mitochondrial dysfunction found in CVDs. In cultured cardiomyocytes, C2 ceramide was found to alter mitochondrial membrane potential and cause mitochondrial fragmentation, leading to apoptosis (57). Increased ceramides and resulting apoptosis were also found in cardiomyocytes of mouse models of various types of cardiomyopathy (94, 116). A mouse model of type 1 diabetic cardiomyopathy showed increased ceramide accumulation in myocardium, as well as diastolic dysfunction (117). In a mouse model of type 2 diabetic cardiomyopathy, CerS2- and CerS5-derived ceramides, including VLC ceramides, led to increased mitophagy and lipotoxic cardiomyocyte hypertrophy (56). In rats, mitochondria from reperfused cardiac tissue contained higher ceramide levels than controls did, specifically in the detergent-resistant portion of the outer mitochondrial membrane (118). This correlation was also found in human subjects with CVD. Myocardial tissue from heart failure patients revealed increased ceramide content as well as reduced PGC-1α expression, which indicates impaired oxidative respiration in mitochondria (113). Across species, from mice to rats to humans, ceramides are strongly linked to CVD through mitochondrial dysfunction.

Interestingly, specific diets may even play a role in ceramide-induced mitochondrial function and cardiomyopathy. Mice that developed diabetic cardiomyopathy due to HFD made from milk fat, rather than from lard, had higher levels of C14 ceramide in cardiac tissue. These mice on milk fat-based HFD also developed cardiac dysfunction more quickly than mice on a lard-based HFD. Treatment with myriocin reversed the previously abnormal electrocardiogram parameters in mice fed a milk fat-based HFD, and it also prevented development of left ventricular and cardiomyocyte hypertrophy (119). Cumulatively, these studies of animal models and patients strongly implicate ceramides in the development and maintenance of CVD.

## Adiponectin and Ceramides

Adiponectin is a prevalent adipokine that acts on many tissues and organs, including the heart, liver, and kidney (120–122). Its actions are correlated with better metabolic health, resulting in improved insulin sensitivity, reduced inflammation, and enhanced cell survival (122, 123). In congenitally leptin-deficient ob/ob mice, which have severe diabetes and metabolic dysfunction (124), adiponectin overexpression was able to reverse their phenotype, restoring normal glucose and insulin levels (125). This dramatic shift underscores the powerful, beneficial role of adiponectin in metabolic health. It has also been shown to improve both alcoholic and non-alcoholic hepatic steatosis (126). Adiponectin promotes expansion of adipose tissue in a benign manner, retaining metabolic health by preventing aberrant lipid accumulation, as occurs in the liver in hepatic steatosis (125, 127, 128).

### Ceramidase Activity of Adiponectin Receptors

After the discovery of adiponectin, the most common adiponectin receptors, AdipoR1 and AdipoR2, were cloned (129). They were later classified as part of the progestin and adiponectin receptor (PAQR) family (130). A fungal member of the PAQR family, Izh2p, was found to be inhibited by drugs affecting sphingolipid metabolism, including myriocin, which inhibits SPT and D-erythro-MAPP, which inhibits ACER (131). Later, discovery of the protein crystal structure and *in vitro* work established that AdipoRs are seven-transmembrane receptors with topology opposite that of GPCRs. These receptors have basal ceramidase activity that is increased by adiponectin binding. They have been shown to bind many anionic, but not neutral, phospholipids and sphingolipids, including species such as ceramide-1-phosphate and dihydroceramide-1-phosphate (132). Moreover, AdipoR2 was purified bound to a molecule of C18 free fatty acid, and a ceramide binding pose was identified by computer simulations. Using fluorophore-labeled C18:0, it was confirmed by fluorescence spectroscopy that ceramide binds AdipoR2 (1, 133). Adiponectin receptors can hydrolyze short ceramides and long ceramides, but C18:0 is hydrolyzed most efficiently. Adiponectin binding increases the basal AdipoR2 ceramidase activity 20-fold as shown by ultra-performance liquid chromatography mass spectrometry (UPLC-MS) (1). While this enzymatic activity is slow, other work has shown that intermembrane enzymes tend to be slower than their soluble counterparts (134). Due to the ceramidase activity of its receptors, adiponectin is hypothesized to have an important role in ceramide metabolism.

Adiponectin receptors have long been a potential target for amelioration of obesity-associated metabolic dysfunction (135), and its intrinsic ceramidase activity makes it a pharmacologic target for therapeutic lowering of ceramides as well. Kadowaki and colleagues developed a candidate drug for this purpose: an orally administered small-molecule agonist of adiponectin receptors 1 and 2 called AdipoRon that binds to both adiponectin receptors at low micromolar concentration (136). This compound has been shown to activate the ceramidase activity of adiponectin receptors, indicating its potential as a ceramide-lowering compound (137, 138).

### FGF21-Adiponectin-Ceramide Axis

Another protein, FGF21, has been implicated in the signaling of adiponectin and ceramides. This protein is known to have antidiabetic properties, as it causes weight loss and improved insulin sensitivity (139). Our group, as well as Lin and colleagues, found that adiponectin secretion in mice is increased by FGF21 (107, 140). We also found that increased FGF21, in mice and cell culture, lowered circulating ceramides, and that mice lacking FGF21 have impaired adiponectin production and increased circulating ceramides. Despite similar circulating FGF21 levels, mice lacking adiponectin and leptin did not respond to FGF21 as compared to ob/ob mice, which significantly lowered hepatic ceramides in response to FGF21. Furthermore, ceramides were increased in DIO mice (107). In another mouse model, an adipocyte-specific knockout of a serine palmitoyl transferase (SPT) subunit resulted in lower ceramide levels and increased FGF21, consistent with this proposed axis (59). In yet another model, depletion of ceramides from suppression of CerS1 globally and in skeletal muscle resulted in improved skeletal muscle insulin sensitivity that was mediated through FGF21 (92).

## Clinical Effects of Ceramide-Induced Dysfunction and Adiponectin

### Insulin Resistance

In female type 2 diabetic children and adolescents, as well as healthy controls, plasma adiponectin and plasma ceramide levels are inversely correlated. These obese diabetic patients had increased levels of ceramide species, including C18:0, C20:0, C22:0, and C24:1, as compared to their lean, non-diabetic counterparts (141). These findings were replicated in adult obese diabetic patients as compared to obese non-diabetic adult controls. Controlling for obesity, diagnosis of type 2 diabetes correlated with increased sphingolipids, dihydroceramides (total, dhC16:0, and dhC24:0), and ceramides (total and C16:0) (59). Dihydroceramides were elevated in patients up to nine years prior to their diabetes diagnosis, demonstrating the value of dihydroceramides as an early sign of metabolic dysfunction (142).

One study analyzed the metabolic health of women with polycystic ovarian syndrome (PCOS), a common gynecological condition linked to metabolic syndrome and insulin resistance (143). In this study, women with PCOS had 25% reduced whole-body insulin sensitivity and 40% lower circulating adiponectin levels. Importantly, their skeletal muscle showed a 300% increase in ceramide levels, further underlining the inverse relationship between circulating adiponectin and ceramide levels (144).

### Cardiovascular Disease

Numerous clinical studies have found correlation between ceramides and CVD. The Dallas Heart Study found that in plasma, adiponectin was inversely correlated with saturated fatty acid chain ceramides only, not unsaturated species. Furthermore, patients with unfavorable lipid profiles were more likely to have short chain ceramides, both saturated and monounsaturated. Importantly, total cholesterol and LDL, two long-accepted metrics of cardiovascular and metabolic health, were strongly positively correlated with increased circulating ceramides (145). In fact, one study found that circulating ceramides may be an early indicator of cardiovascular changes in healthy adults of all ages, and that their plasma levels were inversely correlated with adiponectin levels (115). They are also inversely correlated with the 6-minute walk test, a clinical diagnostic criterion that correlates with aerobic capacity (146). Another study showed that accumulation of ceramides led to arteriolar dilation by inflammatory mediators, while overexpression of ceramidase, exogenous adiponectin, or exogenous sphingosine-1-phosphate (a byproduct of ceramide degradation) led to healthy arteriolar dilation (147). This is important because dysfunction in small vessels often causes or contributes to chronic diseases and their complications, including diabetes mellitus and coronary artery disease (148–151).

## Preclinical Studies of Adiponectin and Ceramides

The clinical studies detailed above collectively show that adiponectin and ceramides levels are negatively correlated, and they strongly suggest a causative link between adiponectin signaling and ceramide metabolism. However, they are unable to probe mechanisms more directly. To this end, studies conducted in cell culture and animal models provide greater insight into the relationship between ceramides and this adipokine.

### Adiponectin and Ceramides

This hypothesized relationship has been investigated with the use of transgenic mice and cells (152). In DIO and ob/ob mice, ceramides are elevated (107) and can be lowered by administration of exogenous adiponectin (108). When adiponectin was overexpressed in adipocytes of DIO mice, the animals remained insulin sensitive with low plasma ceramide levels, despite their increased adiposity. With this overexpression, mice even remained euglycemic when pancreatic beta cells were killed, impairing insulin secretion (108). Even indirect elevation of circulating adiponectin led to increased sphingosine and sphinganine in the liver, demonstrating increased ceramidase activity (153). Conversely, elimination of adiponectin production from adipocytes led to increased ceramide deposition in the liver (108). Specifically, ceramide species C16:0, C18:0, C24:0, and C24:1 were elevated, while ceramide breakdown products sphingosine and sphingosine-1-phosphate (S1P) were lowered (154). Blocking ceramide production with SPT inhibitor myriocin in DIO mice led to increased levels of circulating high molecular weight adiponectin (59). This inverse relationship between adiponectin and ceramides has also been shown in rats (155, 156) and dolphins.

Interestingly, a somewhat different relationship between adiponectin and ceramides is seen in the skin. In these cells, adiponectin plays a beneficial role, aiding in cell differentiation, wound healing, and cell senescence (18, 157–159). Hong et al. found that in response to adiponectin administration, ceramide levels, as well as sphingosine and S1P levels, increased in human epidermal keratinocytes. The authors hypothesized that ceramide levels are elevated either because adiponectin administration also led to increases in overall lipid synthesis through activation of nuclear hormone receptors, or because of inhibition of catabolic action by other ceramidases, which was not assessed (19).

### Adiponectin Receptors and Ceramides

As previously mentioned, adiponectin receptors 1 and 2 are thought to have ceramidase activity. AdipoR1 and AdipoR2 overexpression in adipocytes in gonadal, mesenteric, and subcutaneous adipose tissue (gWAT, mWAT, and sWAT, respectively) increased ceramidase activity (137). Conversely, cultured cells lacking these receptors had impaired ceramidase activity and increased lipid-induced cell death that could not be rescued by adiponectin administration (108). When these receptors are overexpressed in the liver, ceramidase activity increases (108). This hepatic overexpression also leads to lower ceramide species in mWAT and sWAT, indicating communication between these organs (137). In contrast, in their absence, mouse embryonic fibroblasts have impaired ceramidase activity (108). A synthetic adiponectin receptor agonist was developed that can stimulate AdipoR effects in the absence of adiponectin (136), and its injection into wildtype mice showed a comparable increase in ceramidase activity as compared to mice overexpressing AdipoR1 or AdipoR2 in hepatocytes (137).

Exogenous adiponectin administration is able to reverse the diabetic phenotype of ob/ob mice (125). Furthermore, reduction of ceramides has been shown to increase insulin sensitivity. However, the question remained whether specifically the ceramidase action of adiponectin receptors is required to improve insulin sensitivity in leptin-deficient mice. Holland et al. found that overexpression of AdipoR2, but not AC, was able to rescue glucose homeostasis in leptin-deficient mice. Adiponectin receptors lower a larger variety of ceramide species, including deoxyceramides, which are possibly more cytotoxic because of the limited pathways for metabolizing these alanine (rather than serine) derived sphingolipids, than does AC (137).

## Leptin and Ceramides

Leptin is another major adipokine that plays a prominent role in feeding and adipose homeostasis, as well as in reproduction, maintenance of bone mass, immunity, etc. (160, 161). Its release from adipose conveys a satiety signal to the hypothalamus, leading to lower food intake (162). The diabetic phenotype of ob/ob mice is ameliorated by administration of exogenous leptin. However, mice lacking the leptin receptor, known as db/db mice, do not respond to leptin as they cannot detect it (124, 163).

### Leptin Resistance and Ceramides

Leptin resistance is most extreme in the db/db mouse, but in mice and humans, it occurs in DIO as well (163–165). Hyperleptinemia has been shown to be necessary for the development of leptin resistance (166). These high levels of leptin have been shown to increase expression of SOCS3 in the hypothalamus, which blocks transduction of leptin signaling, causing leptin resistance (167, 168). Recently, our group has shown that reversing hyperleptinemia by reducing circulating leptin via genetic and pharmacological means restores hypothalamic sensitivity to leptin, leading to reduced weight gain and increased insulin sensitivity. This intervention leads to increased expression of LepR and POMC in the hypothalamus and subsequent improvement in metabolic phenotype (169, 170). Other research indicates that ceramide levels may play a role in modulating leptin sensitivity as well.

Several studies have directly linked ceramides to leptin resistance. One study showed that administration of myriocin, an inhibitor of *de novo* ceramide synthesis, to obese mice (both DIO and ob/ob) led to reduced levels of leptin expression in epididymal fat. Myriocin treatment had no effect on SOCS3 expression in leptin-deficient ob/ob mice, but it decreased SOCS3 expression in DIO mice. Together, these results indicate that lowering ceramide levels increases leptin sensitivity and that this increased sensitivity cannot occur in the absence of leptin signaling. Furthermore, administration of ceramides to 3T3-L1 adipocytes led to increased SOCS3 expression, further indicating that elevated ceramides induce leptin resistance (60). A recent study replicated this in rats, showing that administration of ceramides induced leptin resistance. Conversely, downregulation of acid sphingomyelinase, which produces ceramide, ameliorated leptin resistance in rats, as indicated by increased expression of leptin receptor and decreased expression of SOCS3 (171).

### Peripheral Effects of Leptin and Ceramides

Several studies indicate that leptin can reduce ceramide levels, but its ability to do so may depend on leptin sensitivity. One study in Sprague-Dawley rats found that exercise-induced lowering of muscle ceramide content, which was initially elevated from HFD, precede any improvement in leptin or insulin sensitivity in skeletal muscle (172). From these data, the authors hypothesize that ceramides may be an important determinant of insulin sensitivity, and the same conclusion could be made regarding ceramides’ impact on leptin sensitivity as well. In another rat model, Wistar rats, leptin caused decreased total ceramide levels in gWAT, as well as decreased expression of genes encoding several ceramide synthesis enzymes, including SPT, LASS2, LASS4, SMPD1, and SMPD2 (173). In Zucker diabetic fatty (ZDF) rats, which have hyperleptinemia and leptin resistance, administration of leptin failed to block upregulation of SPT, resulting in increased ceramide content. However, with increased leptin sensitivity (conferred by delivery of leptin receptor cDNA to islet cells), leptin was able to block SPT mRNA expression (174). This indicates a relationship between leptin and ceramides that is dependent upon leptin sensitivity.

Just as leptin has been shown to reduce ceramides, some studies show that ceramides have a similar, reciprocal regulatory effect on leptin. Treatment of 3T3-L1 pre-adipocytes with ceramide-1-phosphate decreases leptin secretion (175). In DIO mice, which have hyperleptinemia and resultant leptin resistance, treatment with myriocin lead to lower circulating ceramides and decreased gene expression of leptin and SOCS3 in gWAT (60). Reduction of leptin levels has been shown to increase leptin sensitivity, and SOCS3 is known mediator of leptin resistance (167–169, 176). Therefore, reduced expression of leptin and SOCS3 secondary to ceramide reduction may enable increased leptin sensitivity.

However, the relationship between ceramides and leptin is still not entirely clear, as some studies have shown contradictory data. In both the DIO and ob/ob mouse models of obesity, which have high and nonexistent circulating levels of leptin respectively, total plasma and hepatic ceramide levels are increased (108, 177). These studies indicate that regardless of leptin levels, ceramides are increased in models of obesity and diabetes. In gonadal white adipose (gWAT) of leptin-deficient ob/ob mice, while C14 ceramide was increased, total ceramide levels as well as those of C18:1, C24:0, and C24:1 were decreased, showing that ceramides do not respond uniformly to the absence of leptin (177). In adipocytes, knocking out a subunit of SPT (leading to lowered adipose and circulating ceramides), led to lower levels of circulating leptin (59). Therefore, further research is necessary to clarify the relationship between leptin and ceramides, in states of both leptin sensitivity and leptin resistance.

### Cardiovascular Effects of Leptin and Ceramides

Ceramide accumulation and leptin resistance can induce dysfunction in cardiovascular tissue as well. Leptin levels correlate with cardiovascular dysfunction in male patients, independent of degree of obesity and hypertension (178). One possible explanation of this correlation is demonstrated by one study, which showed that cardiac contractile dysfunction was exacerbated by accumulation of ceramides in cardiac muscle, which potentiated the contractile effects of leptin (179). Another study showed that decreasing ceramides in rat aortic endothelial cells, through downregulation of acid sphingomyelinase, improved leptin sensitivity (171). These studies suggest that ceramides contribute to leptin resistance in cardiovascular tissues.

### Central Effects of Leptin and Ceramides

Recent studies have shown that ceramide effects in the hypothalamus contribute to leptin resistance. It has been shown that palmitate, a lipid long known to cross the blood-brain barrier (180) and also induce inflammation (181) does so in the hypothalamus partially through ceramide synthesis (182). Mice subjected to intravenous emulsions of saturated fatty acids had ceramides accumulate in the hypothalamus, indicating a hypothalamic role for ceramide detection and regulation (108). Infusion of a ceramide analog directly into the hypothalamic arcuate nucleus, a brain region that regulates many neuroendocrine functions including feeding (183), of Sprague-Dawley rats suppressed leptin-induced anorectic effects, indicating an inverse relationship between ceramide levels and leptin sensitivity. Physiologic data supports this relationship as well, as fasting (a state of low leptin) increases ceramides, and refeeding (high leptin) decreases ceramides in the arcuate nucleus (2). Conversely, lowering hypothalamic ceramide levels through administration of myriocin increased leptin-induced anorectic effects. In the arcuate nucleus, mRNA expression of SPT, the enzyme driving *de novo* ceramide synthesis, was comparable to expression of AgRP and POMC, which are both crucial to the arcuate’s role in food intake (2). Such significant levels of expression of a key ceramide synthesis enzyme points to an important role for ceramides in this region.

Gao et al. propose that mechanistically, leptin reduces ceramide content through modulation of malonyl-CoA and carnitine palmitoyltransferase 1c (CPT-1c). They hypothesize that leptin induces its anorectic effects in part by increasing malonyl-CoA, which in turn inhibits CPT-1c, leading to decreased *de novo* ceramide synthesis. CPT-1c may act as a transporter of palmitoyl-CoA into the ER, enabling its use as a substrate for ceramide synthesis. Therefore, inhibition of CPT-1c through leptin-induced increases in malonyl-CoA would in turn lower ceramide levels (2). This group found further evidence of ceramide regulation by CPT-1c in hippocampal dendrites, bolstering evidence for this relationship between CPT-1c and ceramides (184).

Another study found that in Zucker rats, a strain known to have leptin resistance, an accumulation of ceramides in the mediobasal hypothalamus led to ER stress and lipotoxicity (185). ER stress has previously been shown to cause leptin resistance (3–5). The authors postulated that ceramides induce ER stress through prevention of proper protein folding. Relief of this ceramide-induced ER stress led to subsequent leptin sensitization, indicating bidirectional control of leptin sensitivity by ceramides (6). However, another study found that central administration of leptin caused decreased ceramide levels in the plasma membrane and Golgi, but not the ER of Wistar rat eWAT (173). Therefore, it is unclear whether ER stress is the true mediator of ceramides’ effect on leptin sensitivity.

## Summary and Future Directions

Over the past few decades, studies have begun to show the significant impact of ceramides and adipokines on metabolic health, particularly in diabetes and CVD. Higher levels of ceramides correlate with low levels of adiponectin as well as with leptin and insulin resistance, indicating that these sphingolipids contribute to dysfunction in cardiovascular, hepatic, and adipose tissue. Cumulatively, these studies suggest that monitoring ceramide levels could allow better assessment of cardiovascular and metabolic disease progression and/or severity, and that ceramides are a possible target for future therapeutic intervention in cardiometabolic pathologies.

## Author Contributions

All authors contributed to the article and approved the submitted version.

## Funding

The authors are supported by US National Institutes of Health (NIH) grants R01-DK55758, RC2-DK118620, P01-DK088761, R01-DK099110 and P01-AG051459 (PS). PS was also supported by an unrestricted research grant from the Novo Nordisk Foundation.

## Conflict of Interest

The authors declare that the research was conducted in the absence of any commercial or financial relationships that could be construed as a potential conflict of interest.
